# Revisiting Formal Copper(III) Complexes: Bridging Perspectives with *Quasi*‐*d* ^*10*^ Configurations

**DOI:** 10.1002/ejic.202200247

**Published:** 2022-08-23

**Authors:** Isaac F. Leach, Remco W. A. Havenith, Johannes E. M. N. Klein

**Affiliations:** ^1^ Molecular Inorganic Chemistry Stratingh Institute for Chemistry University of Groningen Nijenborgh 4 9747 AG Groningen The Netherlands; ^2^ Zernike Institute for Advanced Materials University of Groningen Nijenborgh 4 9747 AG Groningen The Netherlands; ^3^ Ghent Quantum Chemistry Group Department of Chemistry Ghent University Krijgslaan 281 (S3) Ghent 9000 Gent Belgium

**Keywords:** Bonding theory, Computational chemistry, Oxidation states, Population analysis, Transition metals

## Abstract

The formal Cu(III) complex [Cu(CF_3_)_4_]^1−^ has often served as a paradigmatic example of challenging oxidation state assignment – with many reports proposing conflicting descriptions. Here we report a computational analysis of this compound, employing Energy Decomposition Analysis and Intrinsic Bond Orbital Analysis. We present a *quasi‐d* ^*10*^ perspective of the metal centre, resulting from ambiguities in *d‐*electron counting. The implications for describing reactions which undergo oxidation state changes, such as the formal reductive elimination from the analogous [Cu(CF_3_)_3_(CH_2_Ph)]^1−^ complex (Paeth *et al. J. Am. Chem. Soc*. **2019**, 141, 3153), are probed. Electron flow analysis finds that the changes in electronic structure may be understood as a *quasi‐d* ^*10*^ to *d* ^*10*^ transition at the metal centre, rendering this process essentially redox neutral. This is reminiscent of a previously studied formal Ni(IV) complex (Steen *et al*., *Angew. Chem. Int. Ed*. **2019**, *58*, 13133–13139), and indicates that our description of electronic structure has implications for the understanding of elementary organometallic reaction steps.

## Graphical abstract

Lively debates about oxidation states often centre around [Cu(CF_3_)_4_]^−^. Two opposing views have emerged that describe this metal centre as having either a classical or inverted ligand field (implying d^8^ Cu^III^ or d^10^ Cu^I^ configurations, respectively). This perspective explores the Cu^I^/Cu^III^ dichotomy, bridging these views by switching between the localized and delocalized conceptions of bonding.

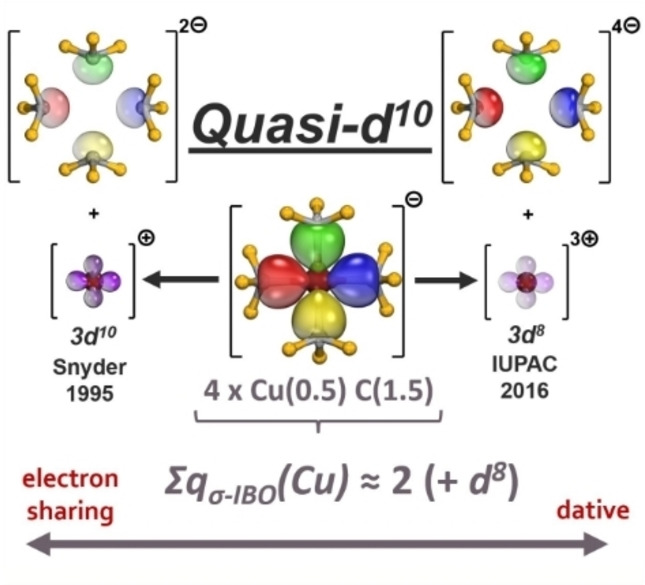

## Oxidation states and d–configurations of TM complexes

The oxidation state (***OS***) formalism is widely acknowledged to be extremely useful, both to classify and group the vast array of chemical compounds and to keep track of the electron count during reactions. Transition metals (TMs) often have partially filled *d*‐shells and there is an intimate connection between the ***OS*** formalism and the *d*‐electron count. Organometallic chemists, as pointed out by Hartwig, commonly use the metal's *d*‐electron count to describe “the number of electrons not involved in the primary metal‐ligand bonding interactions”.^[1]^ As such, the number of metal *d*‐electrons may be used in place of the formal ***OS***. Simple coordination complexes, such as hexaaquacopper(II), are clear‐cut cases where little ambiguity arises thanks to the highly dative bonds formed by the innocent water ligands. However, in organometallic complexes, an increased electron sharing covalent nature of the metal‐to‐ligand bonds is more common.^[2]^ This leads to uncertainty over which nucleus the bonding electrons ‘belong’ to, a shortcoming of the ***OS*** formalism that has been pointed out by Green,^[3]^ who introduced the L‐ (dative), X‐ (electron‐sharing) and Z‐ (inverted dative) ligand types. A classical case of extreme Z‐type bonding between a metal and a ligand, as described by Parkin, is found in transition metal borane complexes.^[4]^ Metal *d*‐orbitals significantly participate in bonding, demonstrating that care has to be taken when assigning *d‐*configurations and ***OS*** s.^[5]^ The resulting grey area, time and time again, renders ***OS*** assignment challenging, where in particularly notorious cases such as transition metals with NO ligands the term noninnocence has been introduced.^[6]^ Especially when reaching the “upper limits”^[7]^ of oxidation state assignments, ambiguity in the metal‐ligand bonding can render simple formal assignments inappropriate. These simple differences, if clearly defined within the ***OS*** formalism, remain useful. The influence of highly covalent bonding can be neglected, as long as the formalism is strictly applied. This sometimes leads to ***OS*** assignments that no longer reflect the behaviour of a transition metal complex. In such instances, a distinction between the formal and physical ***OS***, as pointed out by Wieghardt,^[8]^ may more effectively communicate a compound's properties.

An everlasting example of difficult ***OS*** assignment that sparked much discussion^[9]^ shortly after its first synthesis^[10]^ in the 1990s is [Cu(CF_3_)_4_]^1−^. This collection of seventeen atoms continues to divide the community, lying at the heart of debates around OS assignment. Reports propose either the *d* ^*10*^ Cu(I)[^9b^, ^9c^, ^11^] or *d* ^*8*^ Cu(III)[^9a^, ^12^] extremes, demonstrating the lack of consensus on this issue. In some cases, authors even take different stances within a single article.^[13]^ This molecule nicely demonstrates that while Hartwig's textbook classification of bonding and non‐bonding *d*‐electrons, and its connection to ***OS*** s, is crucial, deeper insight into the electronic structure of a given transition metal's complex is often required. In this article we will revisit the electronic structure of the “quasi square‐planar” complex [Cu(CF_3_)_4_]^1−^,^[9b]^ providing a basis intended to not only clarify the origin of copper's ambiguous ***OS***, but also unify the seemingly disparate perspectives on this molecule. The passionate debates surrounding the electronic structure of this complex have been dubbed the “Oxidation State Wars”.^[13]^ We hope our view provides a compromising solution that satisfies the conflicting parties on this issue.

So, let us begin by outlining the bases for the different ***OS*** assignments for this, at first glance, simple transition metal complex. First and foremost, we have the formal Cu(III) assignment. P. Karel *et al*. provided a much‐needed clarification of the procedure and definition of formal ***OS*** assignments in their recent IUPAC Recommendations report.^[14]^ They define the oxidation state as the charge of an atom “after ionic approximation of its heteronuclear bonds”^[14b]^ i. e. for [Cu(CF_3_)_4_]^1−^, we are instructed to apply the ionic approximation by assigning both the electrons in these heteronuclear bonds to carbon, the more electronegative element. This leaves a formal 3+ charge at the *4s^0^3d* ^*8*^ Cu centre, which we identify as the oxidation state of Cu(III). This is consistent with a square planar geometry, due to a contribution from the lone pair of the CF_3_^‐^ ligands datively donating into the unoccupied Cu *3d*_*x2‐y2*_ orbital. Copper, being a late transition metal with a relatively high electronegativity, is particularly unhappy to accommodate this build‐up of positive charge so, via the charge self‐regulation mechanism described by Raebiger *et al*.,^[15]^ the metal‐ligand bonding assumes an increased degree of electron‐sharing covalency.

## Connecting vocabularies: the spectrum of covalent bonding

**Figure 1:**
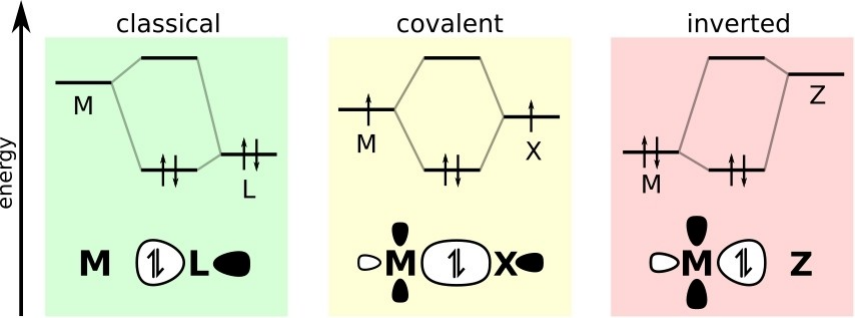
Qualitative molecular orbital diagrams of the CBC ligand‐type bonding scenarios: L‐ (left), X‐ (centre), and Z‐ (right), corresponding to classical, covalent and inverted ligand fields, respectively, as outlined by Lancaster and co‐workers.^[11b]^ Adapted with permission from Ref. [4] Copyright © 2006, American Chemical Society.

As different authors prefer to use different terms to discuss bonding scenarios in organometallic complexes, it is worthwhile to pause and clarify the connections between framings. An alternative approach, that allows for classifications of ligands and ultimately the transition metal's *d*‐electron count, is Green's widely accepted covalent bond classification (CBC) method.^[3]^ This approach describes three types of ligands: L‐type ligands, which act as two‐electron Lewis bases to form dative bonds; X‐type ligands, which both donate and accept one electron to form electron‐sharing bonds; and Z‐type ligands, which act as two‐electron Lewis acids to form ‘inverted’ dative bonds. Parkin showed how these bond types are a function of the relative energies of the metal and ligand orbitals that participate in bonding,^[4]^ shown schematically in Figure 1. Within this formalism, although the nature of the metal‐ligand bonding is distinguished, we still obtain a *d* ^*8*^ configuration of Cu (non‐bonding *d*‐electrons). Curiously, this scheme closely corresponds to the distinctions between classical (Figure 1, left), covalent (Figure 1, centre) and inverted (Figure 1, right) ligand fields, as outlined by Lancaster and co‐workers,^[11b]^ which is rooted in a more spectroscopic conception of bonding. As these bonding scenarios sometimes involve ambiguity in the metal‐ligand bonding featuring sigma symmetry, Hoffmann *et al*.,^[13]^ proposed the use of the term σ‐noninnocence to emphasize that a grey area for ***OS*** assignment has been reached. While this is distinct from the classically noninnocent behaviour of, for example, an NO ligand that now shall be referred to as π‐noninnocence, it has also been observed in formal Ni(IV) complexes by us.^[16]^

How much electron density does Cu in [Cu(CF_3_)_4_]^1−^ recover *via* σ‐noninnocence? Almost exactly two electrons worth. This was first observed in population analyses of calculated wavefunctions by Snyder in 1995,^[9b]^ who raised the question if we might therefore better classify this system as containing a *d* ^*10*^ Cu(I) centre. A total d‐count approaching ten is consistent with Cu X‐ray absorption spectra, simulated and measured by Lancaster and co‐workers,[^11a^, ^11b^] although similar X‐ray spectroscopic data from Cutsail and co‐workers has more recently been suggested to be consistent with a physical *d* ^*8*^ Cu(III) centre engaged in highly (electron‐sharing) covalent bonding.^[12c]^

**Figure 2:**
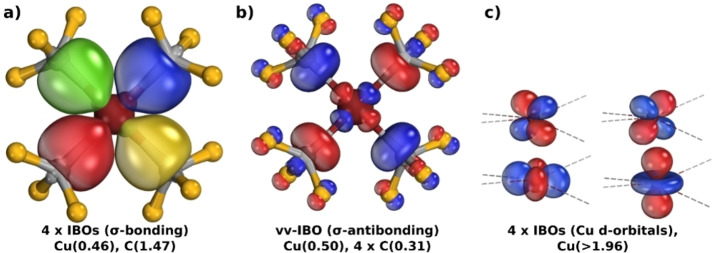
The four occupied IBOs (a) and single virtual valence (vv‐)IBO (b) of the metal‐ligand σ‐space and the four well‐localised Cu *d*‐orbitals (c) in [Cu(CF_3_)_4_]^1−^, showing its intrinsic *d* ^*8*^ configuration, calculated at the PBE0^[22]^/def2‐TZVPP^[23]^//B97‐3c^[24]^level of theory. Note that each of the four σ‐IBOs in (a) is coloured arbitrarily, while blue and red indicate phases in (b, c).

The idea of refraining from using the total *d*‐electron count to assign ***OS*** was employed in the 2011 work by Sit *et al*.,^[17]^ who suggested projecting a calculated wavefunction onto a minimal basis of metal *d*‐functions, allowing the total number of *d*‐electrons to be separated into bonding and non‐bonding components. Only the latter are used for formal oxidation state assignment. In 2013, Knizia noted that Intrinsic Atomic Orbitals (IAOs) may serve as an improved basis for this purpose, as they are pre‐polarised by the molecular environment.^[18]^ Furthermore, projection onto the IAOs is done automatically to remove basis set dependency when producing the Intrinsic Bonding Orbitals (IBOs), which exactly express a calculated wavefunction in chemically convenient terms. As such, once an optimised geometry and wavefunction at (a) chosen level(s) of theory are obtained, orbital localisation is performed, and the *d*‐configuration is obtained *via* simple counting of the number of orbitals with *d*‐symmetry that well localise onto the metal centre. We here term the *d*‐configuration obtained *via* this procedure the *intrinsic d*‐configuration, as it is derived from IBOs. This approach is in line with the spirit of the LOBA method for computational ***OS*** assignment,^[19]^ for which the need for better localised orbitals was recently underlined.^[12a]^ and the more recent OSLO method, which uses a (modified) IBO basis.^[12b]^ For the complex in question, our procedure, conceptually aligned with Sit *et al*.^[17]^ and Hartwig,^[1]^ produces four well‐localised *d‐*orbitals with partial orbital charges at the metal approaching two (Figure 2c) i. e. an intrinsic *d* ^*8*^ configuration. This result supports a Cu(III) assignment, and is in agreement with other computational treatments, EOS,^[20]^ LOBA,^[19]^ and OSLO,^[12b]^ as recently reported by Gimferrer *et al*.,^[12]^ and also at our chosen level of theory (see SI for details). Notably this aligns with the spectroscopic analysis of Cutsail and co‐workers.^[12c]^ What cannot be ignored, however, are the metal‐ligand σ‐IBOs (Figure 2a). Where do they fall on the spectrum of covalent bonding? Do they have significant metal *d*‐character? And if so, at what point does this contribute to the ***OS*** assignment when involved in bonding? This is, indeed, the crux of the issue. The four equivalent σ‐IBOs each have a partial charge distribution of *q(C, Cu)=(1.47, 0.46)e* i. e. the sum of copper's partial σ‐orbital charges, *Σq*_*σ‐IBO*_, is 1.83e, again reflecting the recovery of its lost electron density and indicating that the Cu−CF_3_ bonds each lie between the L‐type (dative) and X‐type (electron‐sharing) extremes. This is reminiscent of Green's X^−^→L rule.^[3a]^ A Löwdin population analysis^[21]^ of the σ‐IBOs reveals each one has a contribution of 14.1 % from the Cu *3d*_*x2‐y2*_ function. If this *d*‐orbital participates so much in the primary metal‐ligand bonding, one would expect to find an antibonding orbital with significant metal *d*‐character. This is precisely what is observed when probing the unoccupied space *via* the virtual valence (vv‐)IBOs.^[16]^ We note here that in the current case of a small, highly symmetric complex, this vv‐IBO is equivalent to the canonical KS‐DFT LUMO. The contribution of the Cu *3d*_*x2‐y2*_ function to the σ‐antibonding vv‐IBO (Figure 2b) is 30.5 %, again *via* Löwdin population analysis, confirming its mixing with the ligand orbitals i. e. rehybridisation into the formed σ‐bonds. In addition to the *3d*_*x2‐y2*_, the Cu *4s* function also participates in the metal‐ligand σ‐framework (Figure S1).

## From orbital to energy analyses

Aside from localised orbital analyses, such as the IBOs presented above, energy decomposition analyses (EDAs) are another powerful tool to interpret the output of quantum calculations. The Morokuma‐Ziegler^[25]^ EDA defines the instantaneous interaction energy (*ΔE_int_*) as the difference between the fully relaxed complex and its separated fragments, frozen in their complex geometries. *ΔE_int_* is then further divided into contributions that can be chemically interpreted e. g. steric interactions are given by the Pauli repulsion term. Here, we focus on the orbital interaction energy (*ΔE_orb_*) as it measures the similarity between the fragment orbitals and the molecular wavefunction. If different choices are made for the fragments, or the fragment states, *ΔE_orb_* can be used to judge the appropriateness of the choice. A small *ΔE_orb_* implies a suitable fragmentation choice, as less energy is ‘released’ (*in silico*) when the constrained fragment orbitals are fully relaxed.^[26]^ For a more detailed discussion of EDA see the reviews by Bickelhaupt & Baerends,^[27]^ and Hopffgarten & Frenking.^[28]^

The EDA results of [Cu(CF_3_)_4_]^1−^, for the interaction between the ensemble of ligands and the single metal ion, are shown in Table 1. The smallest (most favourable) *ΔE_orb_* is found for a *d* ^*10*^ Cu centre (−191.22 kcal mol^−1^), consistent with a +I oxidation state. In contrast, the more oxidised *d* ^*8*^ Cu fragment has the largest *ΔE_orb_* (−923.43 kcal mol^−1^), with the other configurations falling somewhere in between (see Table S1 for additional configurations). These results show that the charge self‐regulation mechanism (discussed above) causes the electron‐sharing nature of the Cu‐CF_3_ bonds to become so dominant that fragmenting into a *3d* ^*10*^ Cu centre (with a 2‐ charge shared over the four trifluoromethyl ligands) is more facile than to a *3d* ^*8*^ Cu centre (with a 4‐ charge on the ligand framework). Despite this configuration arising from significant occupation of the formally empty Cu *3d*_*x2‐y2*_, the *3d* ^*10*^ Cu fragment orbitals are more similar to those of [Cu(CF_3_)_4_]^1−^. As previously discussed, *d*‐orbital population that arises from electron‐sharing covalent bonding is disregarded by the oxidation state formalism, independent of its magnitude. This follows from the ionic approximation, as defined by IUPAC.^[14b]^ To resolve the tension between the origin of this bonding scenario (i. e. from Cu *d* ^*8*^, as seen in its intrinsic configuration, Figure 2c) and its final configuration (closer to Cu *d* ^10^, as seen in the EDA results, Table 1), and the ensuing debates over the Cu ***OS***, we may consider a *quasi‐d* ^*10*^ description of this formal Cu(III) centre. *Quasi‐d* ^*10*^ refers to a bonding scenario in which the total number of *d*‐electrons is 10. These are the eight non‐bonding metal *d*‐electrons (the intrinsic configuration), plus two electrons originating from non‐innocent metal‐ligand σ‐bonding,[^13^, ^29^] where a clear‐cut distinction between metal/ligand centred occupation cannot be made. This label emphasizes the gap between the formal *d* ^*8*^ configuration and the observed electronic structure. Moreover, a distinction from genuine *d* ^*10*^ copper(I) configurations is maintained – which can be easily identified by observation of an intrinsic *d* ^*10*^ configuration. We explore this point further below in the context of a reductive elimination (RE) from a formal Cu(III) centre. Furthermore, this allows for counting of *d*‐electrons arising from electron‐sharing bonding, in contrast to the heuristic oxidation state formalism.

**Table 1:** The orbital interaction energy (*ΔE_orb_*) and instantaneous interaction energy (*ΔE_int_*) in kcal mol^−1^ from EDA calculations of [Cu(CF_3_)_4_]^1−^, fragmenting into (CF_3_)_4_^(n+1)−^ and Cu^n+^ with various *d*‐configurations. The configuration with the smallest *ΔE_orb_* (*s^0^d* ^*10*^) is emphasized in bold.^[a]^

Cu fragment charge [n]	Cu fragment configuration	*ΔE_orb_*	*ΔE_tot_*
3	[Ar](4s)^0^(3d)^8^	−923.43	−2247.72
2	[Ar](4s)^0^(3d)^9^	−350.63	−1044.10
**1**	**[Ar](4s)^0^(3d)^10^**	−**191.22**	−**427.06**
0	[Ar](4s)^1^(3d)^10^	−428.47	−229.00

[a] Calculated at the PBE0/TZ2P//B97‐3c level of theory.

## Quasi‐d^10^ configurations in reactivity

**Figure 3:**
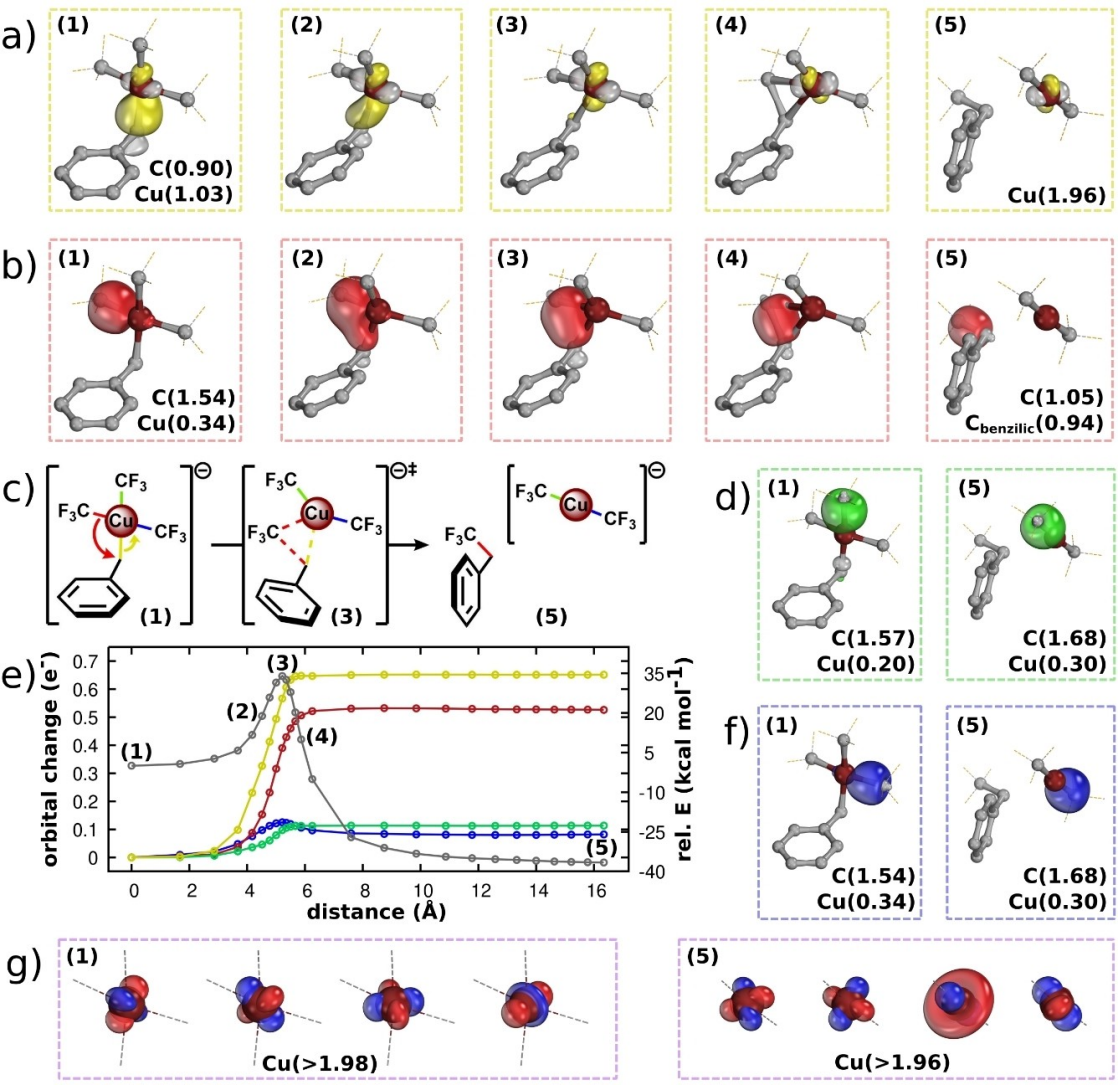
Electron flow analysis of the reductive elimination from [Cu(CF_3_)_3_(CH_2_Ph)]^1−^,^[31]^ showing a quasi‐*d* ^*10*^ to *d* ^*10*^ change in the Cu configuration. The 4 x σ‐IBOs (a, b, d, and f) are shown with their partial charges along selected points **(1)**–**(5)** of the reaction (c). The energy profile of the NEB reaction path,^[32]^ calculated at the PBE0^[22]^/def2‐TZVPP^[23]^//B97‐3c^[24]^ level of theory, as implemented in ORCA 4.2.1,^[33]^ with orbital changes overlayed (e). The intrinsic configurations of Cu in the reactant, *d* ^*8*^, (g, left) and product, *d* ^*10*^ (a and g, right).

We can see how *quasi‐d* ^*10*^ configurations behave during chemical transformations by performing an electron flow analysis^[30]^ of the Csp^3^−Csp^3^ bond‐forming RE from the [Cu(CF_3_)_3_(CH_2_Ph)]^1−^ complex, reported by Paeth *et al*.^[31]^ (Figure 3). It is important to note that the electronic structure of this compound, although different in some respects, can also be described as a *quasi‐d* ^*10*^ configuration, in analogy to that of [Cu(CF_3_)_4_]^1−^. The key difference is that the Cu−C${}_{\mathrm{CH}{}_{2}\mathrm{Ph}}$ bond contains the majority of the electron‐sharing nature, at the expense of the other Cu−CF_3_ bonds. Electron flow analysis consists of tracking the continuous changes in the localised orbitals (the IBOs) along the reaction coordinate, which gives direct insight into changes in bonding and the consequences for the relevant oxidation states. We have previously used this approach to study such reactivity from a formal Ni(IV) complex,^[16]^ and found that the high degree of electron‐sharing covalency in the metal‐ligand σ‐bonds causes the metal centre to be more reduced than its formal ***OS*** would imply. This effectively redox neutral transformation is comparable to the reactivity of the copper complex described here. Let us therefore examine how the related changes occur in [Cu(CF_3_)_3_(CH_2_Ph)]^1−^. During the reaction, the four‐coordinate quasi square‐planar formal Cu(III) centre transforms into a two‐coordinate linear formal Cu(I) centre *via* elimination of trifluoroethyl benzene (Figure 3c). The Cu−C_CH2Ph_ bond of the formal Cu(III) complex (Figure 3a, left) shows strong electron‐sharing covalency as reflected in the partial charge distribution, *q*_*σ‐IBO*_ *(Cu, C)=(1.03, 0.90)*. We identify the electronic structure as *quasi‐d* ^*10*^, as reflected by the gap between its intrinsic *d* ^*8*^ configuration (Figure 3g, left) and the total IAO *d‐*count of *9.40e*. This is corroborated by the EDA, which shows once again that fragmenting to a *d* ^*10*^ centre is most favourable (see Table S2 for details). During the reaction, the σ‐IBO describing the Cu−CF_3_ bond gradually transforms into the newly formed F_3_C−C_CH2Ph_ bond (Figure 3b, red). At the same time, the Cu−C_CH2Ph_ bond is cleaved and morphs into a *d*‐orbital located exclusively at Cu (Figure 3a, yellow), constituting a *d* ^*10*^ configuration in the classical sense. The changes in these two IBOs (Figure 3a, 3b) represent the majority of the electronic redistribution of the reaction, as seen in the magnitude of the IBO orbital changes along the reaction coordinate (Figure 3e). However, two other IBOs, representing the remainder of the metal‐ligand σ‐framework (Figure 3d, Figure 3f, shown in green and blue, respectively), undergo some additional minor reorganisation. The carbon partial charges within these orbitals increase in the reaction – *q*_*σ‐IBO*_ *(C)=(1.57→1.68)* and *q*_*σ‐IBO*_ *(C)=(1.54*→*1.68)*, Figure 3d and 3 f, respectively – representing a slight decrease in electron‐sharing character, consistent with the complete population of the full *d*‐orbital manifold (Figure 3g, left vs. Figure 3a, right, and Figure 3g). The total changes in the summed Cu partial charge (*1.00e*→*0.44e*) and *d*‐count (*9.40e*→*9.73e*) are small compared to the formal 2*e* change that a reductive elimination would suggest, in line with the original NBO analysis by Paeth *et al*.^[31]^ Overall, we can understand the change in electronic structure at the metal centre as a *quasi‐d* ^*10*^ to *d* ^*10*^ transformation, corresponding to a formal change in ***OS*** of Cu(III) to Cu(I). The *quasi‐d* ^*10*^ to *d* ^*10*^ transition well describes this formal RE scenario, where the metal ligand‐bonding has a high degree of electron‐sharing covalency, as the effective ***OS*** changes are minor. The observations described here show how the charge self‐regulation mechanism, previously described in ionic and semi‐conducting crystals,^[15a]^ manifests itself in chemical reactivity.

## Conclusions

Chemistry is sometimes said to be mostly counting, but the devil is in the details – and how we count matters. In the case of [Cu(CF_3_)_4_]^1−^, strict adherence to the ***OS*** formalism requires only non‐bonding *d*‐electrons to be counted, drawing one to the conclusion of a *d* ^*8*^ Cu(III) configuration. On the other hand, if bonding‐electrons are also considered, the total *d*‐count approaches ten due to the degree of electron‐sharing covalency in the Cu−C σ‐bonds, pointing one towards a *d* ^*10*^ Cu(I) (inverted ligand field) configuration. This ambiguity has resulted in much debate over the most appropriate description. We share our description of this copper centre as *quasi‐d* ^*10*^, to emphasize the difference between the formal and effective *d*‐counts, while maintaining a distinction from genuine *d* ^*10*^ Cu(I).

**Figure 4:**
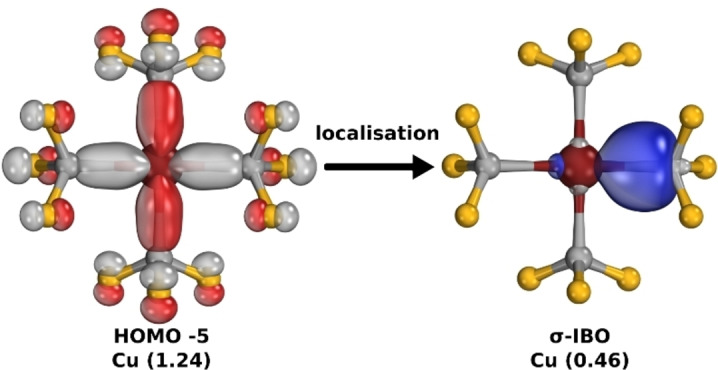
Delocalised (HOMO −5, left) and localised (σ‐IBO, right) pictures of the metal‐ligand σ‐bonding in [Cu(CF_3_)_4_]^1−^, with the partial orbital charges of copper, calculated with PBE0^[22]^/def2‐TZVPP^[23]^//B97‐3c.^[24]^

The question of whether [Cu(CF_3_)_4_]^1−^ should be considered to have an inverted ligand field or a copper(III) ***OS*** boils down to the delocalised vs. localised conceptions of chemical bonding, which are both valid views.^[34]^ Computationally, the delocalised picture (Figure 4, left) is more closely related to the energetic basis (e. g. from the Fock operator) and can thus be more easily connected to (X‐ray) spectroscopy, which probes energetic transitions. The localised picture (Figure 4, right) is more closely related to the position basis and thus more easily connects to our chemical intuitions e. g. of localised two‐centre, two‐electron bonds in Lewis structures. Of course, while the individual orbitals do change considerably, the Slater determinants constructed from the set of all occupied orbitals (localised or not) are mathematically equivalent, as they are related via unitary transformations. The IBO localisation procedure is a way to flip between these conceptually opposed views, helping us build bridges between them.

In studying the formal reductive elimination from [Cu(CF_3_)_3_(CH_2_Ph)]^1−^, we found this reaction can be understood as a transition from a *quasi‐d* ^*10*^ to *d* ^*10*^ copper configuration, a finding that has direct relevance to our understanding of elementary organometallic reaction steps – which so often play key roles in catalytic cycles. We hypothesize that while sometimes difficult to describe, a strong *give and take* in electron‐sharing bonding character, and the consequently minor changes in effective oxidation states and electronic reorganisation throughout catalytic cycles, may be required to achieve efficient catalysis. This may lead to a distinction in the reactivity of transition metals between those that involve genuine changes in oxidation states, and those that are effectively redox neutral.

## Disclaimer

The opinions expressed in this publication are the view of the author and do not necessarily reflect the opinions or views of the *European Journal of Inorganic Chemistry*, the Publisher, Chemistry Europe, or the affiliated editors.

## Conflict of interest

The authors declare no conflict of interest.

1

## Biographical Information

*Johannes E. M. N. Klein received his B.Sc. degree from the Universität Dortmund, Ger‐many (renamed to Technische Universität Dortmund) and his M.Sc. from University College Dublin, Ireland in chemistry. Following research stays at Harvard University, USA (group of Prof. Dr. Tobias Ritter) and the University of York, UK (group of Prof. Dr. Richard J. K. Taylor) he obtained his doctorate degree from the Universität Stuttgart, Germany in 2014 under the tutelage of Prof. Dr. Bernd Plietker. After a postdoctoral stay at the University of Minnesota, the USA in the group of Prof. Dr. Lawrence Que, Jr., he was in 2017 appointed as an assistant professor at the Stratingh Institute for Chemistry at the University of Groningen, The Netherlands. His research interests lie at the interface of organic, inorganic and computational chemistry. (Photo @ Sylvia Germes)*

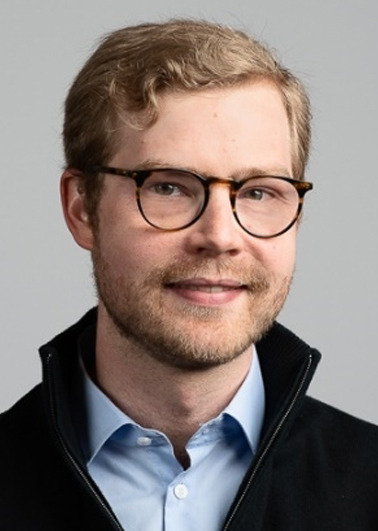

## Supporting information

As a service to our authors and readers, this journal provides supporting information supplied by the authors. Such materials are peer reviewed and may be re‐organized for online delivery, but are not copy‐edited or typeset. Technical support issues arising from supporting information (other than missing files) should be addressed to the authors.

Supporting Information

## Data Availability

The data that support the findings of this study are available in the supplementary material of this article.
