# Supplementary material for: Revisiting Formal Copper(III) Complexes: Bridging Perspectives with Quasi‐d 10 Configurations
Source: Eur J Inorg Chem. 2022 Aug 23;2022(27):e202200247. doi: 10.1002/ejic.202200247 (PMC9804752; doi:10.1002/ejic.202200247)
Supplement: Supplementary file 1 — Supporting Information [file EJIC-2022-0-s001.pdf]

European Journal of Inorganic Chemistry

Supporting Information

Revisiting Formal Copper(III) Complexes: Bridging Perspectives with *Quasi*-d^{10} Configurations

Isaac F. Leach, Remco W. A. Havenith, and Johannes E. M. N. Klein*

Table of Contents

**Computational details**	S1
**Coordinates of optimised geometries**	S2
**Virtual localized orbital of $[Cu(CF_3)_4]^{1-}$**	S5
**Energy Decomposition Analysis**	S5
**$[Cu(CF_3)_4]^{1-}$**	S5
**Deformation densities**	S6
**$[Cu(CF_3)_3(CH_2Ph)]^{1-}$**	S6
**Computational Oxidation States of $[Cu(CF_3)_4]^{1-}$**	S7
**EOS**	S7
**LOBA**	S8
**EOS of the formal reductive elimination from $[Cu(CF_3)_3(CH_2Ph)]^{1-}$**	S8
**Data plotted in Figure 3e**	S9
**Frontier MOs for $[Cu(CF_3)_4]^-$**	S10
**References**	S10

Computational details

Geometry optimisations of $[Cu(CF_3)_4]^{n-}$

Geometries of $[Cu(CF_3)_4]^{n-}$ (*n=1, 2, 3*) were optimised (*Opt*) using the efficient composite DFT method B97-3c.[1] The nature of each stationary point was confirmed by numerical vibrational analyses (*NumFreq*). No symmetry was enforced during the optimisation. All geometry optimisations were performed in ORCA 4.2.1,[2] using a finer than default integration grid (*Grid5 NoFinalGrid*) and an energetic convergence criterion of 10^{-8} Hartree (*TightSCF*). The electronic structures used for the IBO analyses were obtained *via* single point energy calculations performed at the optimised geometries using the PBE0 functional[3] and the def2-TZVPP basis set.[4] The resolution of identity approximation (*RIJCOSX*)[5] was employed in conjunction with Weigend's universal fitting basis set (*def2/J)*.[6] Localisation and visualization of the IBOs and virtual valence (vv-)IBOs was performed in an unreleased version of IboView (v2018).[7] All orbitals isosurfaces are shown such that 80% of the corresponding electron density is enclosed. In addition, Löwdin population analyses[8] were performed on both the canonical σ anti-bonding orbitals and the (*IAOIBO)* localised orbitals as implemented in ORCA 4.2.1.[2]

Geometry optimisations of the reductive elimination

Geometries of $[Cu(CF_3)_3(CH_2Ph)]^{1-}$ (RE reactant) and $[Cu(CF_3)_2{\bullet}(CF_3CH_2Ph)]^{1-}$ (RE product complex) were optimised (*TightOpt*) using the efficient composite DFT method B97-3c.[1] The nature of each stationary point was confirmed by analytical vibrational analyses (*Freq*). All geometry optimisations were performed in ORCA 4.2.1,[2] using a finer than default integration grid (*Grid5 NoFinalGrid*) and an energetic convergence criterion of 10^{-8} Hartree (*TightSCF*). No symmetry was enforced during the optimisation. These optimised stationary points were used as input for a fixed end point climbing image nudged elastic band[9] (*NEB-CI*) reaction path with 24 intermediate images, using with the B97-3c method. The same grid and convergence criteria (*Grid5 NoFinalGrid TightSCF*) were used, as in the geometry optimizations.

The electronic structures used for the electron flow analyses were obtained *via* single point energy calculations performed on the NEB geometries using the PBE0 functional[3] and the def2-TZVPP basis set.[4] The resolution of identity approximation (*RIJCOSX*)[5] was employed in conjunction with Weigend's universal fitting basis set (*def2/J)*,[6] also with a fine integration grid (*Grid5 NoFinalGrid*) and an energetic convergence criterion of 10^{-8} Hartree (*TightSCF*). Localization and visualization of the IBOs and virtual valence (vv-)IBOs was performed in an unreleased version of IboView (v2018).[7] All orbitals isosurfaces are shown such that 80% of the corresponding electron density is enclosed.

The NEB-CI implementation in ORCA[10] ensures the optimized reaction path well approximates the minimum energy path between the RE reactant $[Cu(CF_3)_3(CH_2Ph)]^{1-}$ and the RE product complex $[Cu(CF_3)_2{\bullet}(CF_3CH_2Ph)]^{1-}$. This is confirmed by using the converged climbing image (the highest energy structure from the NEB reaction path, RE-CI) as input for a transition state optimization (*OptTS*), which easily converged to an optimized saddle point (RE-TS) with a single imaginary frequency of -260.90 cm^{-1} (corresponding to $F_3C\text{-}C_{CH2Ph}$ bond formation) and a B97-3c Gibbs free energy (G) of 27.7 kcal mol^{-1}, relative to the RE reactant $[Cu(CF_3)_3(CH_2Ph)]^{1-}$, close to $G_{RE\text{-}CI}$ (32.3 kcal mol^{-1}). The all-atom root mean squared deviation of RE-CI and RE-TS is 0.92 Å and the partially formed bond ($F_3C\text{-}C_{CH2Ph}$) distances are 2.05 and 2.07 Å, respectively.

Energy Decomposition Analysis

To further probe the electronic structure, and in particular the Cu *d*-configuration, Morokuma-Ziegler Energy Decomposition Analysis (EDA)[11] was performed in the Amsterdam Density Functional (ADF) suite of the AMS 2020 package.[12] These calculations were performed at the B97-3c optimized *n=1* geometry from ORCA, and employed the PBE0 functional[3] in combination with the triple-ζ TZ2P basis set.[13] No frozen core approximation was made. Scalar relativistic effects were given *via* a ZORA Hamiltonian.[14] Numerical quality was defined with the *Good* keyword. For each EDA calculation only two fragments were defined: 1) the metal centre, Cu^{n+} and 2) the entire remaining ligand framework $(CF_3)_4^{(n+1)-}$. The optimised coordinates were reoriented such that the Cu-CF_3 bonds lay along the *xy* axes, providing optimal overlap between the ligand and metal ($3d_{x2\text{-}y2}$) orbitals. Although no symmetry was enforced during the complex and ligand calculations, local D_{4h} symmetry was applied to the metal fragment to enable specification of the $4s^0 3d^8$, $4s^0 3d^9$ and $4s^1 3d^{10}$ electronic configuration *via* the *IrrepOccupations* keyword.

Coordinates of optimized geometries

$[Cu(CF_3)_4]^{1-}$	
17<br>charge -1 multiplicity 1<br>Cu 0.00000257816712 -0.00000214986889 0.00000033722950<br>C 1.95281419638495 0.41233970042022 0.26721166958806<br>F 2.60839057686119 -0.55258709368116 0.99086269012213<br>F 2.66833968720836 0.56511655718934 -0.88545902818196<br>F 2.16175741338455 1.55820399300402 0.99369742145031<br>C -0.41241305763957 1.95282702324569 -0.26697523131815<br>F -0.56485183225149 2.66828308067538 0.88578281049430<br>F 0.55230409560353 2.60844358953870 -0.99086924409579<br>F -1.55848765206111 2.16181879223078 -0.99311547491395<br>C 0.41235378702975 -1.95274167344556 -0.26771577147735<br>F 0.56506206243448 -2.66858227079392 0.88476817899346<br>F -0.55252990466735 -2.60811917898824 -0.99160313073995<br>F 1.55826098004895 -2.16148716421210 -0.99419187091482<br>C -1.95275452239460 -0.41242638216937 0.26747920263199<br>F -2.60817658234216 0.55226363325131 0.99158445053387<br>F -2.66851930887675 -0.56482254044914 -0.88509261015713<br>F -2.16155231688986 -1.55852771794704 0.99363420275544	$E_{B97\text{-}3c}$ = -2991.217434008938<br>Thermal correction to G* = 0.00716715<br><br>$(E_{PBE0/def2\text{-}TZVPP})_{n=1}$ = -2990.182410610395<br>$(E_{PBE0/def2\text{-}TZVPP})_{n=2}$ = -2990.088859619806<br>$(E_{PBE0/def2\text{-}TZVPP})_{n=3}$ = -2989.740646636399<br><br>*1 atm 298K (B97-3c)

$[Cu(CF_3)_4]^{2-}$	
17<br>charge -2 multiplicity 2<br>Cu 0.00026376580525 -0.00002620430065 0.00006855508216<br>C 1.93271256692002 0.37910348427012 -0.66896924983633<br>F 2.91934476705977 0.58098423380957 0.29845997993968<br>F 2.49549566787422 -0.62849556306240 -1.47438546992410<br>F 2.06976202831782 1.51578237458042 -1.48763193081796<br>C -0.38173655694427 1.93511601185351 0.65981498304184<br>F 0.62298691990131 2.50129547613488 1.46647293220198<br>F -0.58042438295037 2.91788470732780 -0.31217948269224<br>F -1.52113089240687 2.07517469640651 1.47414898429631<br>C 0.38161090414765 -1.93002239931274 0.67483188060501<br>F -0.62184554060472 -2.48834364863103 1.48849937064390<br>F 0.57644232870793 -2.92071311843731 -0.28989781506196<br>F 1.52299955071227 -2.06530192846738 1.48714573203893<br>C -1.93239612859628 -0.38426437463672 -0.66575956739923<br>F -2.91935599097360 -0.57517023771473 0.30360040645168<br>F -2.49387466851545 0.61588859836397 -1.48132165652598<br>F -2.07084741245470 -1.52889579818382 -1.47290669604368	$E_{B97\text{-}3c}$ = -2991.140661236489<br>Thermal correction to G* = -0.00240149<br><br>$(E_{PBE0/def2\text{-}TZVPP})_{n=2}$ = -2990.111042585588<br><br>*1 atm 298K (B97-3c)

$[Cu(CF_3)_4]^{3-}$	
17<br>charge -3 multiplicity 1<br>Cu 0.00012847694869 -0.00023281854046 0.00033914943943<br>C 1.71619938677899 0.25985129089134 -1.27012293330765<br>F 3.01399089254042 0.41875626821086 -0.64982981332164<br>F 2.03841200515475 -0.78646694022505 -2.21650068957847<br>F 1.74618362887547 1.39757311890444 -2.16403298295883<br>C -0.25290206212859 1.74654783612895 1.22898881277570<br>F 0.84105428517304 2.15076399217422 2.08585647595602<br>F -0.51845821534680 3.01245867874397 0.58002879990187<br>F -1.32428047140530 1.75570292715545 2.20160911060716<br>C 0.29124355017361 -1.72382617101238 1.25334820319842<br>F -0.77630756832535 -2.11164605551644 2.15016632763065<br>F 0.53755840703563 -3.00173263500206 0.62062344074395<br>F 1.39146619120526 -1.71512689742528 2.19333924986937	$E_{B97\text{-}3c}$ = -2990.921721755746<br>Thermal correction to G* = -0.01234375<br><br>$(E_{PBE0/def2\text{-}TZVPP})_{n=3}$ = -2989.881908614977

C -1.75387180713019 -0.28303274545930 -1.21211604696149<br>F -3.03166330897335 -0.43485756833231 -0.54984469500049<br>F -2.10702886064887 0.74859157673490 -2.16324951453512<br>F -1.80921843092744 -1.43388336643083 -2.08784973745886	*1 atm 298K (B97-3c)

$[Cu(CF_3)_4]^{1-}$<br>Reoriented along xy axes, for the EDA	
17<br>charge -1 multiplicity 1<br>Cu 0.00000000 0.00000000 0.00000000<br>C -1.99598187 0.00000326 -0.26637802<br>F -2.43834053 -1.07942265 -0.99004467<br>F -2.72715268 0.00142752 0.88659834<br>F -2.43747826 1.07810407 -0.99247951<br>C 0.00013388 1.99585334 0.26734582<br>F 0.00097431 2.72758151 -0.88527695<br>F -1.07892712 2.43785816 0.99177242<br>F 1.07859878 2.43700277 0.99311770<br>C 0.00013388 -1.99585152 0.26734582<br>F -0.00185324 -2.72758395 -0.88527317<br>F 1.07991289 -2.43785661 0.99070043<br>F -1.07761163 -2.43699487 0.99419012<br>C 1.99572233 0.00000406 -0.26831211<br>F 2.43737446 1.07943500 -0.99240158<br>F 2.72801106 -0.00142594 0.88395382<br>F 2.43651891 -1.07808925 -0.99485123	

$[Cu(CF_3)_3(CH_2Ph)]^{1-}$<br>RE reactant	
27<br>charge -1 multiplicity 1<br>Cu -1.52780548866146 -0.09045565051159 0.35098560852578<br>C -2.07939059820706 -0.66213537869682 2.22862322587399<br>C -1.73519366507633 -1.98392662950780 -0.31091134209538<br>C -1.71187541608362 1.85109805000235 0.86345485681337<br>F -2.88197082246861 -2.57263260720755 0.15876579571642<br>F -1.83310101819733 -2.17125691886214 -1.68500139202721<br>F -0.70858246267944 -2.81478771062377 0.06319326566996<br>F -3.44252983727262 -0.70815999709026 2.39879978038836<br>F -1.61848599575779 0.15800250249034 3.23299079115960<br>F -1.62758424668577 -1.90761381048441 2.60086028107674<br>F -1.80048773229920 2.77721498729248 -0.16943051272719<br>F -2.85579754922508 2.08756786160150 1.58323339379982<br>F -0.67964743434892 2.31918541166360 1.63818994899221<br>C -0.58468582309420 0.42981831795276 -1.37175490987853<br>H -0.91508176585411 -0.26550872892257 -2.12851726390197<br>H -0.89537547653324 1.43735777937528 -1.60345106196057<br>C 0.84083807637887 0.31736041977957 -1.06019780102486<br>C 1.54308293936120 -0.88040779280958 -1.26774913834320<br>C 1.56489972941083 1.40411447714762 -0.54468166524862<br>C 2.89257808196717 -0.98371202774384 -0.98008647871119<br>H 1.00898024142853 -1.73778604384187 -1.65196908904464<br>C 2.91434349224756 1.29961038469925 -0.25721835933557<br>H 1.04778296408175 2.33534451035531 -0.36212223900436<br>C 3.59135903331809 0.10537049419039 -0.47329109685136<br>H 3.40424332671932 -1.92346084796001 -1.15099452314441<br>H 3.44308991953716 2.15740659062738 0.14121710259335<br>H 4.64639752799430 0.02239235708441 -0.24293717731051	$E_{B97\text{-}3c}$ = -2924.515170347238<br>Thermal correction to G* = 0.10688820<br><br>*1 atm 298K (B97-3c)

$[Cu(CF_3)_2{\bullet}(CF_3CH_2Ph)]^{1-}$<br>RE product complex	
27<br>charge -1 multiplicity 1<br>Cu -0.94998812160848 1.67519310365559 1.91667478391870<br>C -0.38456434037031 0.78232954104963 3.55641950803648<br>C -1.56569965506297 -1.82532974169408 -2.11592983459363<br>C -1.60045889958327 2.63942227104321 0.35271711130999<br>F -1.52903257243834 -2.87328685128476 -1.25666141623525<br>F -2.85060894504378 -1.72814760141582 -2.55848914374966<br>F -0.81616375157280 -2.18099660271362 -3.19622800573762<br>F -1.01828123753509 1.22116885615959 4.71781117495792<br>F 0.97338245849036 0.89889665225293 3.87296629954307<br>F -0.59164347786297 -0.60784330721532 3.59286332755807<br>F -0.73490049560389 2.70503335139756 -0.74490441174510<br>F -2.77973118659171 2.12555997124058 -0.21381058585640<br>F -1.92094790342620 3.97671707030483 0.58046683748366<br>C -1.10707225022173 -0.53480984477881 -1.48738318346904<br>H -1.22002350526064 0.24562461474289 -2.23654134245821<br>H -1.80703445380018 -0.29482907545813 -0.69133569530970<br>C 0.29720039667732 -0.57195587772635 -0.96102462854558<br>C 1.38081711929962 -0.32776717585529 -1.79915832043788<br>C 0.53094928432534 -0.81504375212196 0.38791854136332<br>C 2.67417074271513 -0.33765979296779 -1.30072922138750<br>H 1.20863193923215 -0.11778853504477 -2.84730953347908<br>C 1.82317168019585 -0.81577715770797 0.89254554328586<br>H -0.29813962948218 -0.98873364547129 1.05826150951968<br>C 2.89718944371963 -0.57839546720904 0.04885112474512	$E_{B97\text{-}3c}$ = -2924.573460208840<br>Thermal correction to G* = 0.10492021

H	3.50727951544286	-0.13657191696978	-1.96278739624587
H	1.97592039885079	-0.96823361727267	1.95115680659832
H	3.90557744651551	-0.56677546893936	0.44364015093031

*1 atm 298K (B97-3c)

RE-TS

27

charge -1 multiplicity 1

Cu	-1.15714342943621	0.06091257081167	0.55569312504380
C	-0.63989739431319	-1.14554656665001	2.07671865847337
C	-2.14742757945591	-1.32458964623474	-0.84291803348977
C	-1.85461468702362	1.96934127823261	0.62743453708458
F	-3.08384000827152	-1.49103015636348	0.14034088073593
F	-2.92242769728788	-1.07348175147331	-1.95405235741957
F	-1.58555947107879	-2.54699188203376	-1.03707616197933
F	-1.60444410354026	-1.29093988181540	3.06887141734872
F	0.47743812098779	-0.68143569026806	2.77579712754832
F	-0.29117498845420	-2.47398123363740	1.78518020937873
F	-1.90485662395056	2.69216464036687	-0.60244354423624
F	-3.16415555258681	2.07005435156955	1.08036484988235
F	-1.15769886745744	2.85235585913461	1.45064096025282
C	-0.67317112518463	-0.07063018556391	-1.56249946697697
H	-0.68000209999276	-0.81433908819666	-2.34596666073397
H	-1.16782068297066	0.84065268137081	-1.86195153989103
C	0.65353278050848	0.07190856298504	-0.95742787495726
C	1.46279777620008	-1.05501933518969	-0.74773857575393
C	1.15692245293700	1.33239918292041	-0.59946851836861
C	2.72216847552880	-0.92647099885108	-0.19303396080788
H	1.07076911916702	-2.03380407440925	-0.98460324729964
C	2.42718210297850	1.45460479319415	-0.06338380665065
H	0.54018612777264	2.20581243501544	-0.74445217100194
C	3.21215192116670	0.32856414907728	0.14537401841278
H	3.32004478491565	-1.81009140728588	-0.01141068605429
H	2.79596989221953	2.43376814563274	0.21295084599080
H	4.19507075662225	0.42581324766146	0.58905997546889

E_{B97-3c} = -2924.467960958464

Thermal correction to G* = 0.10377443

*1 atm 298K (B97-3c)

RE-CI

27

charge -1 multiplicity 1, NOTE this is the 8th image (the climbing image) from the NEB and is therefore NOT a stationary point

Cu	-1.54277738262163	0.24948029412846	0.66235981960407
C	-1.29978255991963	-0.52864816268380	2.49453659581080
C	-1.70889246950802	-1.63655821254195	-0.61235254173688
C	-2.14992118343162	2.17924201129870	0.46603081999426
F	-2.73002852713209	-1.93982463601956	0.25578710751840
F	-2.30137608921435	-1.92829886147054	-1.83168415814587
F	-0.74753042975924	-2.56296452387985	-0.42881927760829
F	-2.48767855592412	-0.55092294353087	3.22364200116117
F	-0.44485576653048	0.23755370162317	3.29024287621381
F	-0.80407024560976	-1.82152147301661	2.65440091662735
F	-1.93435720552862	2.79881792262616	-0.79582868625002
F	-3.50782255260483	2.37953187176118	0.67351424581360
F	-1.54750621110896	3.07797041489110	1.34134289157857
C	-0.64549624587445	-0.06511603176494	-1.38115116741912
H	-0.78583979524596	-0.58344240148305	-2.31576537768519
H	-1.14167479893656	0.89400939897501	-1.42814336224383
C	0.75747876184037	-0.04043174129877	-0.97192331490230
C	1.67169115605517	-0.90691317830773	-1.58443098545171
C	1.23961932451874	0.84190289915742	0.00394496336750
C	3.01542379039788	-0.87924579663158	-1.25868943672465
H	1.31325978373437	-1.60435994395030	-2.33103821863848
C	2.58268749789811	0.85464140283163	0.34303671813098
H	0.55893279621322	1.52187425415696	0.49765217808358
C	3.47758357483154	0.00217644106784	-0.28833127504065
H	3.70472362460592	-1.54886933356170	-1.75829961538949
H	2.93010256046784	1.53889911492946	1.10583821333349
H	4.52810714838718	0.02101751269415	-0.02587193000109

E_{B97-3c} = -2924.460929161690

Thermal correction to G* = 0.10405935

*1 atm 298K (B97-3c)

Virtual localized orbital of $[Cu(CF_3)_4]^{1-}$

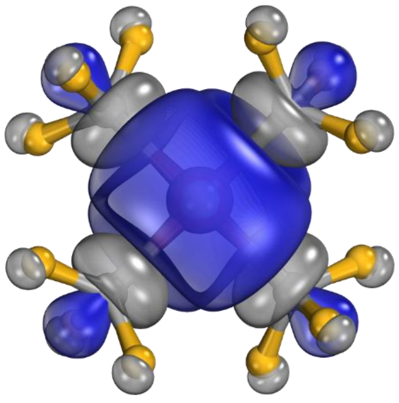

C(1.42) Cu(0.13)

Figure S1: Virtual valence (vv-)IBO and its partial charge distribution of $[Cu(CF_3)_4]^{n-}$ with n=1//n=1, showing the participation of the Cu *4s* orbital in bonding.

Energy Decomposition Analysis

$[Cu(CF_3)_4]^{1-}$

To further probe the electronic structure of $[Cu(CF_3)_4]^{1-}$, and in particular the Cu *d* configuration, Morokuma-Ziegler Energy Decomposition Analysis (EDA)[11] was performed in the Amsterdam Density Functional (ADF) suite of the AMS 2020 package.[12] These calculations were performed at the B97-3c optimised *n=1* geometry from ORCA (please see ‘Computational details’ above for more details), and employed the PBE0 functional[3] in combination with the triple-ζ TZ2P basis set.[13] No frozen core approximation was made. Scalar relativistic effects were given *via* a ZORA Hamiltonian.[14] Numerical quality was defined with the *Good* keyword. For each EDA calculation only two fragments were defined: 1) the metal centre, Cu^{n+} and 2) the entire united ligand framework $[(CF_3)_4]^{(n+1)-}$.

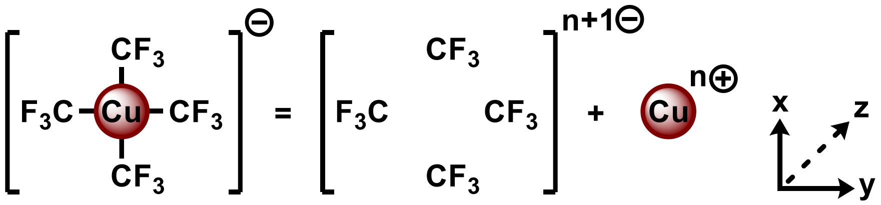

Figure S2: The $[Cu(CF_3)_4]^{1-}$ complex (left), alongside its ligand (centre), metal (right) fragments and axes orientation (far right) as defined for the EDA calculations. The Cu fragment charge, *n*, was varied to achieve the desired Cu *d* configuration (see Table S1).

EDA calculations require specification of the fragments comprising a molecule as input. We chose to fragment $[Cu(CF_3)_4]^{1-}$ into ligand and metal (Figure S2, centre and right, respectively). D_{4h} symmetry (an approximate point group of the *pseudo* square planar complex) was imposed on the metal fragment to ensure alignment between the Cu-C bonding axes and the lobes of the Cu $3d_{x2-y2}$ orbital, whose occupations was controlled *via* the *Irrepoccupations* keyword. The Cu atom defined the origin of the new axes. No symmetry was imposed during the ligand fragment and full complex calculations. The various Cu configurations were specified by changing the fragment charges, spins, and orbital occupations, as summarized in Table S1. For all entries with a non-zero number of unpaired

electrons (Cu), unrestricted fragment orbitals were used. For a more detailed discussion of EDA, the curious reader is directed towards the reviews by Bickelhaupt & Baerends,[15] and Hopffgarten & Frenking.[16]

Table S1: The various Cu^{n+} fragment states used in the EDA calculations of $[Cu(CF_3)_4]^{1-}$. Along with the instantaneous interaction energy (ΔE_{int}) the orbital interaction energy (ΔE_{orb}), the quasiclassical Coulomb interaction (ΔE_{elstat}) and repulsive Pauli exchange (ΔE_{Pauli}) terms are also given. All energies are in kcal mol^{-1}. The entry with the smallest ΔE_{orb} has been emphasized in bold.

**Entry**	**Cu fragment configuration**	Ligand charge, -(*n+1*)	Metal charge, *n*	Number of unpaired electrons (Cu)	ΔE_{int}	ΔE_{orb}	ΔE_{elstat}	ΔE_{Pauli}
#1	$[Ar](4s)^0(3d)^8(3d_{x2-y2})^0$	-4	+3	0	-2247.72	-923.43	-1545.32	221.03
#2	$[Ar](4s)^{1\alpha}(3d)^6(3d_{z2})^{1\beta}(3d_{x2-y2})^0$	-4	+3	0	-2596.31	-1454.22	-1592.73	450.65
#3	$[Ar](4s)^{1\alpha}(3d)^6(3d_{z2})^{1\alpha}(3d_{x2-y2})^0$	-4	+3	2	-2614.80	-1538.52	-1545.91	469.63
#4	$[Ar](4s)^{1\alpha}(3d)^6(3d_{xy})^{1\beta}(3d_{x2-y2})^0$	-4	+3	0	-2673.34	-1536.31	-1597.17	460.14
#5	$[Ar](4s)^{1\alpha}(3d)^6(3d_{xy})^{1\alpha}(3d_{x2-y2})^0$	-4	+3	2	-2691.52	-1622.60	-1550.67	481.75
#6[a]	$[Ar](4s)^{1\alpha}(3d)^6(3d_{xz}3d_{yz})^{1\beta}(3d_{x2-y2})^0$	-4	+3	0	-2657.06	-1518.37	-1594.16	455.47
#7[a]	$[Ar](4s)^{1\alpha}(3d)^6(3d_{xz}3d_{yz})^{1\alpha}(3d_{x2-y2})^0$	-4	+3	2	-2675.86	-1603.12	-1547.44	474.71
#8	$[Ar](4s)^0(3d)^8(3d_{x2-y2})^{1\alpha}$	-3	+2	1	-1044.10	-350.63	-908.83	215.37
#9	$[Ar](4s)^{1\alpha}(3d)^8(3d_{x2-y2})^0$	-3	+2	1	-1299.88	-808.38	-968.96	477.47
**#10**	$\mathbf{[Ar](4s)^0(3d)^{10}}$	**-2**	**+1**	**0**	**-427.06**	**-191.22**	**-463.75**	**227.91**
#11	$[Ar](4s)^{1\alpha}(3d)^8(3d_{x2-y2})^{1\beta}$	-2	+1	0	-486.15	-457.35	-517.88	489.09
#12	$[Ar](4s)^2(3d)^8(3d_{x2-y2})^0$	-2	+1	0	-704.00	-880.35	-576.55	752.90
#13	$[Ar](4s)^2(3d)^8(3d_{x2-y2})^0$	-2	+1	0	-704.00	-880.35	-576.55	752.90
#14[b]	$[Ar](4s)^{1\alpha}(3d)^{10}$	-1	0	1	-229.00	-428.47	-346.44	545.91
#15[c]	$[Ar](4s)^{1\alpha}(3d)^{10}$	-1	0	1	-220.84	-401.27	-346.51	526.95
#16	$[Ar](4s)^2(3d)^{10}$	0	-1	0	-327.13	-618.60	-508.89	800.35

[a] In general, fractional orbital occupations were avoided but this is not possible for the two dimensional irreducible representation of D_{4h} (E_g), so here the $3d_{xz}$ & $3d_{yz}$ orbitals have occupations = *0.5e*
[b] When no constraints were enforced, this variational solution had two frontier orbitals with occupations = *0.5e*
[c] When no fractional occupations were allowed, this solution was obtained – with only a small difference in ΔE_{orb}

Deformation densities

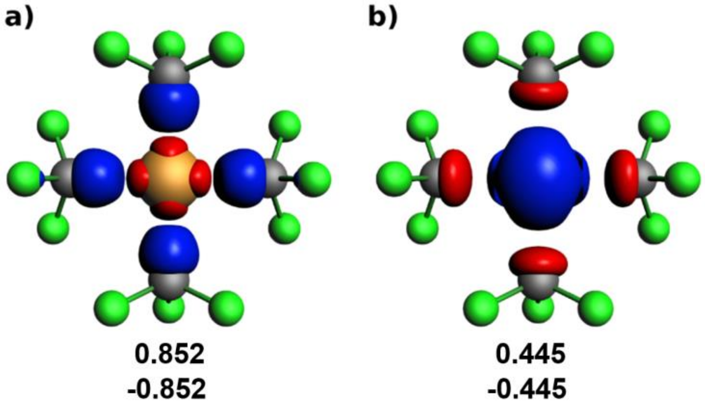

Figure S3: ETS-NOCV pair deformation densities from a) σ-backdonation from the metal $3d_{x2-y2}$ to the ligand and b) σ-donation from the ligand to the metal *4s* orbital. Contributions to the ΔE_{orb} in the EDA -94.4 and -31.8 kcal mol^{-1}, respectively. Corresponding NOCV eigenvalues pairs are given in a.u. The metal fragment was prepared in the $[Ar](4s)^0(3d)^{10}$ state. Isosurfaces drawn at 0.0055 in *AMSview.*

$[Cu(CF_3)_3(CH_2Ph)]^{1-}$

Table S2: The various Cu^{n+} fragment states used in the EDA calculations of $[Cu(CF_3)_3(CH_2Ph)]^{1-}$. Along with the instantaneous interaction energy (ΔE_{int}) the orbital interaction energy (ΔE_{orb}), the quasiclassical Coulomb interaction (ΔE_{elstat}) and repulsive Pauli exchange (ΔE_{Pauli}) terms are also given. All energies are in kcal mol^{-1}. The entry with the smallest ΔE_{orb} has been emphasized in bold.

Entry	Cu fragment configuration	Ligand charge, -(*n*+1)	Metal charge, *n*	Number of unpaired electrons (Cu)	ΔE_{int}	ΔE_{orb}	ΔE_{elstat}	ΔE_{Pauli}
#1	$[Ar](4s)^0(3d)^8(3d_{x2-y2})^0$	-4	3	0	-2185.47	-1021.62	-1382.64	218.79
#2	$[Ar](4s)^0(3d)^8(3d_{x2-y2})^{1\alpha}$	-3	2	1	-1019.95	-383.72	-841.61	205.38
**#3**	**$[Ar](4s)^0(3d)^8(3d_{x2-y2})^2$**	**-2**	**1**	**0**	**-419.59**	**-193.73**	**-445.76**	**219.90**
#4	$[Ar](4s)^{1\alpha}(3d)^8(3d_{x2-y2})^2$	-1	0	1	-197.96	-359.94	-316.68	478.66

Computational Oxidation States of $[Cu(CF_3)_4]^{1-}$

EOS

The Effective Oxidative State (EOS) method,[17] developed by P. Salvador and co-workers in 2015 allows for OSs to be determined from calculated wavefunctions. It employs the topological fuzzy Voronoi cells (TFVC),[18] which efficiently approximate Bader's topological basins from the Quantum Theory of Atoms in Molecules,[19] in order to partition the molecular space into atomic regions. Once an optimized geometry is obtained and a wavefunction is computed, the EOS of each of the fragments chosen by the user is computed, along with a reliability index, R, that formally ranges from 0-100%, R=50% indicates degeneracy of two assignments and R<50% indicates "that the assignation of the electrons has not followed an *aufbau* principle".[17] The R value allows the user to try different fragmentation schemes to see which is more appropriate for a given system. Gimferrer *et al.* reported the EOS of Cu in $[Cu(CF_3)_4]^-$ to be +3, with R=51.7%,[20] at the ωB97X-V[21]/def2-TZVP[4] level of theory. The EOSs of copper at our chosen level of theory, PBE0/def2-TZVPP//B97-3c, are shown for several fragmentation schemes (Table S3). We note that our results agree closely with Gimferrer *et al.* only if each $-CF_3$ moiety is treated as a separate fragment (entry 4). This fragmentation scheme seems to bias EOS(Cu) towards +3, perhaps because each $-CF_3$ moiety cannot accept half an electron, as is necessary to agree with Snyder's original d^{10} Cu(I) assignment. If, instead, all four trifluormethyl groups are treated as a united ligand framework (entry 1), EOS(Cu) = +1 with a greatly increased reliability index (R=85.2%). The sensitivity of EOS(Cu) to the choice of fragmentation scheme demonstrates, once again, the ambiguity of copper's OS in this molecule and the need for greater flexibility in our understanding of its electronic structure.

Table S3: Effective oxidation states of copper in $[Cu(CF_3)_4]^{1-}$, calculated at the PBE0/def2-TZVPP//B97-3c level of theory, for several fragmentation schemes, as implemented in a developer version of IboView (v2018).

Entry	1	2	3	4
**Fragmentation scheme**	CF_3<br>F_3C–Cu–CF_3<br>CF_3<br>*united*	CF_3<br>F_3C–Cu–CF_3<br>CF_3<br>*trans*	CF_3<br>F_3C–Cu–CF_3<br>CF_3<br>*cis*	CF_3<br>F_3C–Cu–CF_3<br>CF_3<br>*separated*
**# fragments**	**2**	**3**	**3**	**5**
**EOS(Cu)**	**+1**	**+1**	**+1**	**+3**
**R (%)**	**85.2**	**50.0**	**50.0**	**50.5**

LOBA

In the same work, Gimferrer *et al.*[20] also compute the oxidation state of Cu in $[Cu(CF_3)_4]^{1-}$ within the Localised Orbital Bonding Analysis (LOBA) scheme,[22] developed by Head-Gordon an co-workers. Their result, Cu(III), obtained again at the ωB97X-V/def2-TZVP level of theory, agrees with our calculations performed at the PBE0/def2-TZVPP//B97-3c level. LOBA requires specification of a localization scheme, a method for the population analysis, a method for the orbital compositions and a percentage threshold to assign electrons of localized orbitals to atomic centres. We chose to use Pipek-Mezey localized orbitals,[23] a Mulliken population analysis,[24] the Hirshfeld method for the orbital compositions,[25] as implemented in the opensource program Multiwfn 3.7.[26] A threshold of 50% was specified. The same level of theory was use as our other calculations (PBE0/def2-TZVPP//B97-3c) calculated in Gaussian 16, Revision B.01,[27] using the *PBE1PBE* keyword and the basis set specified manually using the Basis Set Exchange.[28]

EOS of the formal reductive elimination from $[Cu(CF_3)_3(CH_2Ph)]^{1-}$

The electron flow analysis of the reductive elimination from $[Cu(CF_3)_3(CH_2Ph)]^{1-}$ (Figure 3), reported by Paeth *et al.*,[29] can be complemented by the application of the Effective Oxidation State (EOS)[17] approach from P. Salvador and co-workers (briefly discussed above). The results (Table S4) show that EOS(Cu) = +1 throughout the reaction, with a reliability index of R=58% for the RE reactant $[Cu(CF_3)_3(CH_2Ph)]^{1-}$ and R=(63%, 100%) for the RE product complex $[Cu(CF_3)_2{\bullet}(CF_3CH_2Ph)]^{1-}$ (depending on if the trifluoroethyl benzene moiety is treated a single fragment). This parallels our previous observations that EOS(Ni) remained constant throughout a formal reductive elimination,[30] and further justifies consideration of such reactions as effectively redox neutral.

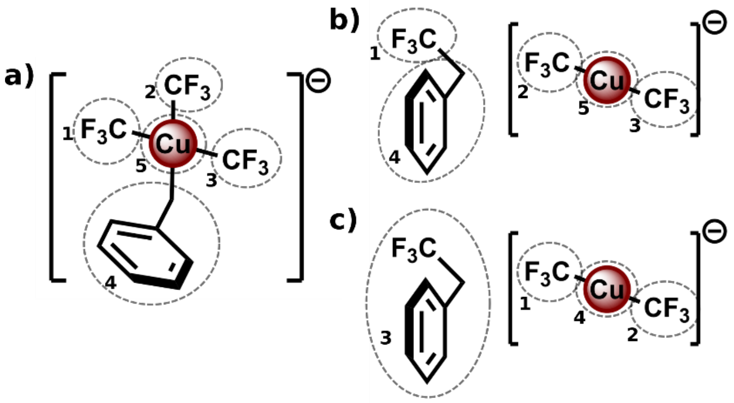

Figure S4: Chosen fragments for a) the reactant and b-c) the product of the reductive elimination from a formal Cu(III) centre (Figure 3c), reported by Paeth *et al*.[29]

Table S5: EOS results, as implemented in IboView v2018, and the reliability indices of the assignment, for the fragment schemes defined above (Figure S2), calculated at the PBE0/def2-TZVPP//B97-3c level of theory.

Fragmentation scheme	(Fragment)$_{Fragment\ \#}$					
	**$(CF_3)_{\#1}$**	**$(CF_3)_{\#2}$**	**$(CF_3)_{\#3}$**	**$(CH_2Ph)_{\#4}$**	**$(Cu)_{\#5}$**	**R (%)**
**a)**	**-1**	**-1**	**-1**	**+1**	**+1**	**57.9**
**b)**	**-1**	**-1**	**-1**	**+1**	**+1**	**63.1**
Fragmentation scheme	**(Fragment)$_{Fragment\ \#}$**					**R (%)**
	**$(CF_3)_{\#1}$**	**$(CF_3)_{\#2}$**	**$(CF_3CH_2Ph)_{\#3}$**	**$(Cu)_{\#4}$**	**/**	
**c)**	**-1**	**-1**	**0**	**+1**	**/**	**99.9**

Data plotted in Figure 3e

Table S5: The frame (image) numbers, distance along the reaction path (as defined in ORCA 4.2.1),[2,10] SCF energy (E), relative energy (ΔE) and changes in IBOs ($|q_i(s)-q_i(0)|$)[7b] (calculated *via* PBE0/def2-TZVPP single point calculations on the B97-3c optimised NEB-CI path). The colours and symbols (a, b, d, and f) of $|q_i(s)-q_i(0)|$ correspond to the orbital labels in Figure 3.

Frame #	Distance along NEB (Å)	E (Hartree)	ΔE (kcal mol^{-1})	$\|q_i(s)-q_i(0)\|$			
				a	b	d	f
0	0.000	-2924.51517	0.00	0.000000	0.000000	0.000000	0.000000
1	1.673	-2924.51409	0.68	0.002981	0.004361	0.000780	0.009249
2	2.864	-2924.51091	2.67	0.023502	0.010882	0.006271	0.021093
3	3.673	-2924.50596	5.78	0.098078	0.036881	0.021834	0.047475
4	4.171	-2924.49650	11.71	0.230398	0.087248	0.035998	0.075945
5	4.517	-2924.48504	18.91	0.325911	0.152767	0.045779	0.097408
6	4.780	-2924.47391	25.89	0.412579	0.231112	0.060644	0.112415
7	5.002	-2924.46493	31.53	0.493130	0.315580	0.079499	0.121641
8 **(CI)**	5.214	-2924.46093	34.04	0.565953	0.389349	0.097072	0.124931
9	5.355	-2924.46328	32.56	0.605903	0.428206	0.105467	0.123214
10	5.502	-2924.47075	27.87	0.632841	0.459624	0.110070	0.118828
11	5.666	-2924.48302	20.18	0.645686	0.485278	0.111828	0.112549
12	5.872	-2924.49927	9.98	0.647086	0.505860	0.112289	0.105923
13	6.253	-2924.52315	-5.01	0.646685	0.521611	0.112603	0.096510
14	7.595	-2924.55830	-27.06	0.650104	0.529942	0.113124	0.085853
15	8.740	-2924.56470	-31.08	0.651215	0.531842	0.113224	0.083935
16	9.850	-2924.56826	-33.31	0.651485	0.531512	0.113065	0.082227
17	10.877	-2924.57007	-34.45	0.651356	0.530162	0.112786	0.080940
18	11.779	-2924.57095	-35.00	0.651247	0.528979	0.112589	0.080277
19	12.588	-2924.57150	-35.35	0.651173	0.528135	0.112555	0.079997
20	13.327	-2924.57199	-35.66	0.651158	0.527590	0.112636	0.079983
21	14.003	-2924.57248	-35.96	0.651162	0.527228	0.112777	0.080167
22	14.630	-2924.57287	-36.21	0.651279	0.526953	0.112924	0.080508
23	15.220	-2924.57318	-36.40	0.651302	0.526698	0.113033	0.080931
24	15.787	-2924.57338	-36.53	0.651296	0.526451	0.113111	0.081369
25	16.343	-2924.57346	-36.57	0.651266	0.526192	0.113176	0.081872

Frontier MOs for $[Cu(CF_3)_4]^-$

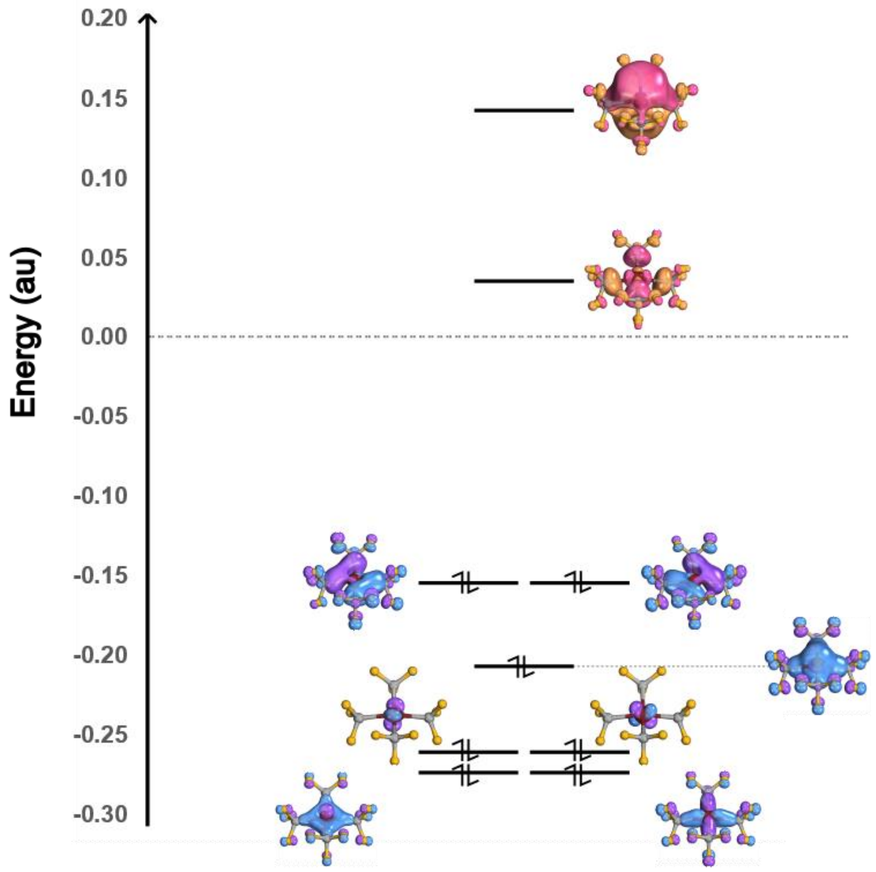

Figure S5: The canonical frontier molecular orbitals for $[Cu(CF_3)_4]^-$, calculated with PBE0/def2-TZVPP//B97-3c.

References

[1] J. G. Brandenburg, C. Bannwarth, A. Hansen, S. Grimme, *J. Chem. Phys.* **2018**, *148*, 064104.

[2] a) F. Neese, *WIREs: Comput. Mol. Sci.* **2012**, *2*, 73-78; b) F. Neese, *WIREs: Comput. Mol. Sci.* **2018**, *8*, e1327.

[3] C. Adamo, M. Cossi, V. Barone, *J. Mol. Struct.: THEOCHEM* **1999**, *493*, 145-157.

[4] F. Weigend, R. Ahlrichs, *Phys. Chem. Chem. Phys.* **2005**, *7*, 3297-3305.

[5] F. Neese, F. Wennmohs, A. Hansen, U. Becker, *Chem. Phys.* **2009**, *356*, 98-109.

[6] F. Weigend, *Phys. Chem. Chem. Phys.* **2006**, *8*, 1057.

[7] a) G. Knizia, *J. Chem. Theory Comput.* **2013**, *9*, 4834-4843; b) G. Knizia, J. E. M. N. Klein, *Angew. Chem. Int. Ed.* **2015**, *54*, 5518-5522.

[8] P.-O. Löwdin, *Physical Review* **1955**, *97*, 1474-1489.

[9] a) H. Jónsson, G. Mills, K. W. Jacobson, in *Classical and Quantum Dynamics in Condensed Phase Simulations*, pp. 385-404; b) G. Mills, H. Jónsson, *Phys. Rev. Lett.* **1994**, *72*, 1124-1127; c) G. Mills, H. Jónsson, G. K. Schenter, *Surf. Sci.* **1995**, *324*, 305-337; d) G. Henkelman, B. P. Uberuaga, H. Jónsson, *J. Chem. Phys.* **2000**, *113*, 9901-9904.

[10] V. Ásgeirsson, B. O. Birgisson, R. Bjornsson, U. Becker, F. Neese, C. Riplinger, H. Jónsson, *J. Chem. Theory Comput.* **2021**, *17*, 4929-4945.

[11] a) K. Kitaura, K. Morokuma, *Int. J. Quantum Chem.* **1976**, *10*, 325-340; b) T. Ziegler, A. Rauk, *Inorg. Chem.* **1979**, *18*, 1755-1759; c) T. Ziegler, A. Rauk, *Inorg. Chem.* **1979**, *18*, 1558-1565.

[12] G. te Velde, F. M. Bickelhaupt, E. J. Baerends, C. Fonseca Guerra, S. J. A. van Gisbergen, J. G. Snijders, T. Ziegler, *J. Comput. Chem.* **2001**, *22*, 931-967.

[13] E. Van Lenthe, E. J. Baerends, *J. Comput. Chem.* **2003**, *24*, 1142-1156.

[14] a) E. v. Lenthe, E. J. Baerends, J. G. Snijders, *J. Chem. Phys.* **1993**, *99*, 4597-4610; b) E. van Lenthe, E. J. Baerends, J. G. Snijders, *J. Chem. Phys.* **1994**, *101*, 9783-9792; c) E. van Lenthe, J. G. Snijders, E. J. Baerends, *J. Chem. Phys.* **1996**, *105*, 6505-6516; d) E. van Lenthe, R. van Leeuwen, E. J. Baerends, J. G. Snijders, *Int. J. Quantum Chem* **1996**, *57*, 281-293; e) E. van Lenthe, A. Ehlers, E.-J. Baerends, *J. Chem. Phys.* **1999**, *110*, 8943-8953.

[15] F. M. Bickelhaupt, E. J. Baerends, in *Rev. Comput. Chem.*, **2000**, pp. 1-86.

[16] M. v. Hopffgarten, G. Frenking, *WIREs: Comput. Mol. Sci.* **2012**, *2*, 43-62.

[17] E. Ramos-Cordoba, V. Postils, P. Salvador, *J. Chem. Theory Comput.* **2015**, *11*, 1501-1508.

[18] P. Salvador, E. Ramos-Cordoba, *J. Chem. Phys.* **2013**, *139*, 071103.

[19] R. F. W. Bader, *Acc. Chem. Res.* **1985**, *18*, 9-15.

[20] M. Gimferrer, J. Van Der Mynsbrugge, A. T. Bell, P. Salvador, M. Head-Gordon, *Inorg. Chem.* **2020**, *59*, 15410-15420.

[21] N. Mardirossian, M. Head-Gordon, *Phys. Chem. Chem. Phys.* **2014**, *16*, 9904.

[22] A. J. W. Thom, E. J. Sundstrom, M. Head-Gordon, *Phys. Chem. Chem. Phys.* **2009**, *11*, 11297.

[23] J. Pipek, P. G. Mezey, *J. Chem. Phys.* **1989**, *90*, 4916-4926.

[24] R. S. Mulliken, *J. Chem. Phys.* **1955**, *23*, 1833-1840.

[25] F. L. Hirshfeld, *Theor. Chim. Acta* **1977**, *44*, 129-138.

[26] a) L. Tian, C. Feiwu, **2011**; b) T. Lu, F. Chen, *J. Comput. Chem.* **2012**, *33*, 580-592; c) T. Lu, F.-W. Chen, *Acta Physico-Chimica Sinica* **2012**, *28*, 1-18.

[27] M. J. Frisch, G. W. Trucks, H. B. Schlegel, G. E. Scuseria, M. A. Robb, J. R. Cheeseman, G. Scalmani, V. Barone, G. A. Petersson, H. Nakatsuji, X. Li, M. Caricato, A. V. Marenich, J. Bloino, B. G. Janesko, R. Gomperts, B. Mennucci, H. P. Hratchian, J. V. Ortiz, A. F. Izmaylov, J. L. Sonnenberg, Williams, F. Ding, F. Lipparini, F. Egidi, J. Goings, B. Peng, A. Petrone, T. Henderson, D. Ranasinghe, V. G. Zakrzewski, J. Gao, N. Rega, G. Zheng, W. Liang, M. Hada, M. Ehara, K. Toyota, R. Fukuda, J. Hasegawa, M. Ishida, T. Nakajima, Y. Honda, O. Kitao, H. Nakai, T. Vreven, K. Throssell, J. A. Montgomery Jr., J. E. Peralta, F. Ogliaro, M. J. Bearpark, J. J. Heyd, E. N. Brothers, K. N. Kudin, V. N. Staroverov, T. A. Keith, R. Kobayashi, J. Normand, K. Raghavachari, A. P. Rendell, J. C. Burant, S. S. Iyengar, J. Tomasi, M. Cossi, J. M. Millam, M. Klene, C. Adamo, R. Cammi, J. W. Ochterski, R. L. Martin, K. Morokuma, O. Farkas, J. B. Foresman, D. J. Fox, Wallingford, CT, **2016**.

[28] B. P. Pritchard, D. Altarawy, B. Didier, T. D. Gibson, T. L. Windus, *J. Chem. Inf. Model.* **2019**, *59*, 4814-4820.

[29] M. Paeth, S. B. Tyndall, L.-Y. Chen, J.-C. Hong, W. P. Carson, X. Liu, X. Sun, J. Liu, K. Yang, E. M. Hale, D. L. Tierney, B. Liu, Z. Cao, M.-J. Cheng, W. A. Goddard, W. Liu, *J. Am. Chem. Soc.* **2019**, *141*, 3153-3159.

[30] J. S. Steen, G. Knizia, J. E. M. N. Klein, *Angew. Chem. Int. Ed.* **2019**, *58*, 13133-13139.
